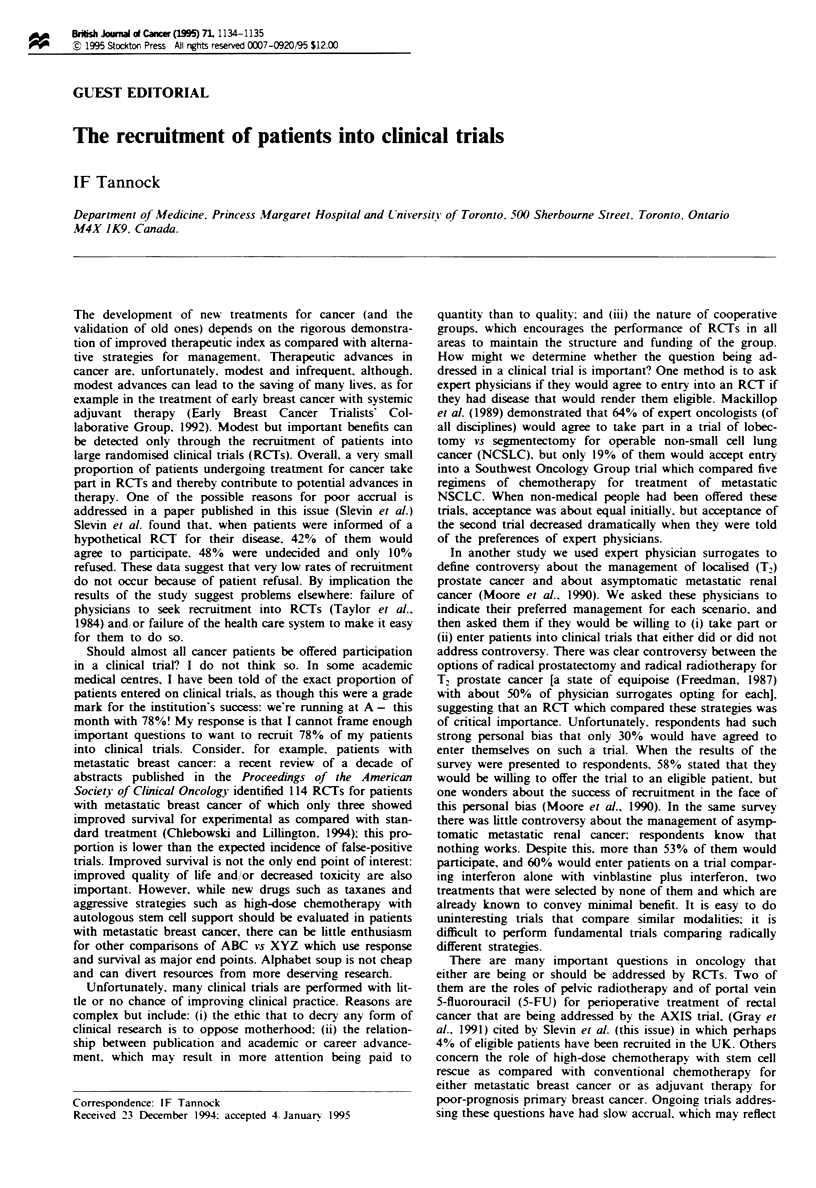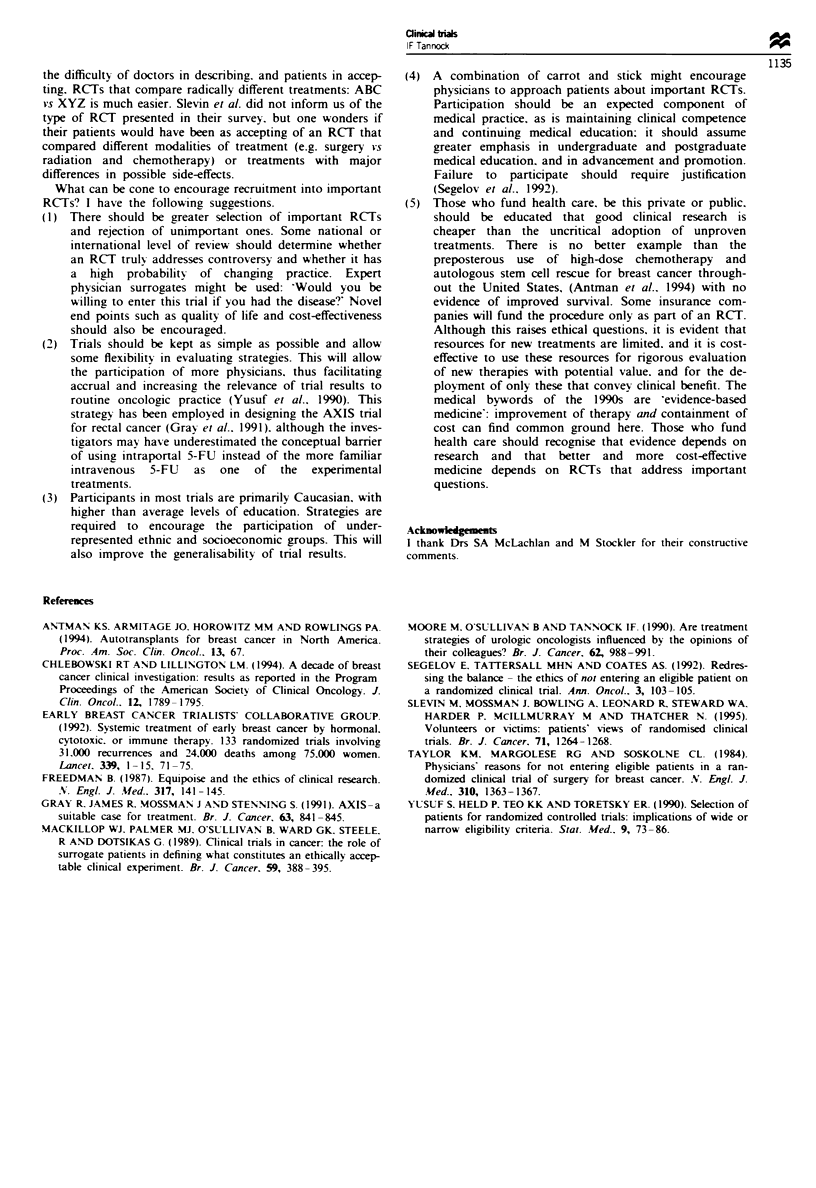# The recruitment of patients into clinical trials.

**DOI:** 10.1038/bjc.1995.221

**Published:** 1995-06

**Authors:** I. F. Tannock


British Journal of Cancer (1995) 71, 1134–1135

© 1995 Stockton Press All rights reserved 0007-0920/95 $12.00

GUEST EDITORIAL

### The recruitment of patients into clinical trials

IF Tannock

*Department of Medicine, Princess Margaret Hospital and University of Toronto, 500 Sherbourne Street, Toronto, Ontario M4X 1K9, Canada.*

The development of new treatments for cancer (and the validation of old ones) depends on the rigorous demonstration of improved therapeutic index as compared with alternative strategies for management. Therapeutic advances in cancer are, unfortunately, modest and infrequent, although, modest advances can lead to the saving of many lives, as for example in the treatment of early breast cancer with systemic adjuvant therapy (Early Breast Cancer Trialists' Collaborative Group, 1992). Modest but important benefits can be detected only through the recruitment of patients into large randomised clinical trials (RCTs). Overall, a very small proportion of patients undergoing treatment for cancer take part in RCTs and thereby contribute to potential advances in therapy. One of the possible reasons for poor accrual is addressed in a paper published in this issue (Slevin *et al.*) Slevin *et al.* found that, when patients were informed of a hypothetical RCT for their disease, 42% of them would agree to participate, 48% were undecided and only 10% refused. These data suggest that very low rates of recruitment do not occur because of patient refusal. By implication the results of the study suggest problems elsewhere: failure of physicians to seek recruitment into RCTs (Taylor *et al.*, 1984) and/or failure of the health care system to make it easy for them to do so.

Should almost all cancer patients be offered participation in a clinical trial? I do not think so. In some academic medical centres, I have been told of the exact proportion of patients entered on clinical trials, as though this were a grade mark for the institution's success: we're running at A – this month with 78%! My response is that I cannot frame enough important questions to want to recruit 78% of my patients into clinical trials. Consider, for example, patients with metastatic breast cancer: a recent review of a decade of abstracts published in the *Proceedings of the American Society of Clinical Oncology* identified 114 RCTs for patients with metastatic breast cancer of which only three showed improved survival for experimental as compared with standard treatment (Chlebowski and Lillington, 1994); this proportion is lower than the expected incidence of false-positive trials. Improved survival is not the only end point of interest: improved quality of life and/or decreased toxicity are also important. However, while new drugs such as taxanes and aggressive strategies such as high-dose chemotherapy with autologous stem cell support should be evaluated in patients with metastatic breast cancer, there can be little enthusiasm for other comparisons of ABC *vs* XYZ which use response and survival as major end points. Alphabet soup is not cheap and can divert resources from more deserving research.

Unfortunately, many clinical trials are performed with little or no chance of improving clinical practice. Reasons are complex but include: (i) the ethic that to decry any form of clinical research is to oppose motherhood; (ii) the relationship between publication and academic or career advancement, which may result in more attention being paid to quantity than to quality; and (iii) the nature of cooperative groups, which encourages the performance of RCTs in all areas to maintain the structure and funding of the group. How might we determine whether the question being addressed in a clinical trial is important? One method is to ask expert physicians if they would agree to entry into an RCT if they had disease that would render them eligible. Mackillop *et al.* (1989) demonstrated that 64% of expert oncologists (of all disciplines) would agree to take part in a trial of lobectomy *vs* segmentectomy for operable non-small cell lung cancer (NCSLC), but only 19% of them would accept entry into a Southwest Oncology Group trial which compared five regimens of chemotherapy for treatment of metastatic NSCLC. When non-medical people had been offered these trials, acceptance was about equal initially, but acceptance of the second trial decreased dramatically when they were told of the preferences of expert physicians.

In another study we used expert physician surrogates to define controversy about the management of localised ($T_2$) prostate cancer and about asymptomatic metastatic renal cancer (Moore *et al.*, 1990). We asked these physicians to indicate their preferred management for each scenario, and then asked them if they would be willing to (i) take part or (ii) enter patients into clinical trials that either did or did not address controversy. There was clear controversy between the options of radical prostatectomy and radical radiotherapy for $T_2$ prostate cancer [a state of equipoise (Freedman, 1987) with about 50% of physician surrogates opting for each], suggesting that an RCT which compared these strategies was of critical importance. Unfortunately, respondents had such strong personal bias that only 30% would have agreed to enter themselves on such a trial. When the results of the survey were presented to respondents, 58% stated that they would be willing to offer the trial to an eligible patient, but one wonders about the success of recruitment in the face of this personal bias (Moore *et al.*, 1990). In the same survey there was little controversy about the management of asymptomatic metastatic renal cancer; respondents know that nothing works. Despite this, more than 53% of them would participate, and 60% would enter patients on a trial comparing interferon alone with vinblastine plus interferon, two treatments that were selected by none of them and which are already known to convey minimal benefit. It is easy to do uninteresting trials that compare similar modalities; it is difficult to perform fundamental trials comparing radically different strategies.

There are many important questions in oncology that either are being or should be addressed by RCTs. Two of them are the roles of pelvic radiotherapy and of portal vein 5-fluorouracil (5-FU) for perioperative treatment of rectal cancer that are being addressed by the AXIS trial, (Gray *et al.*, 1991) cited by Slevin *et al.* (this issue) in which perhaps 4% of eligible patients have been recruited in the UK. Others concern the role of high-dose chemotherapy with stem cell rescue as compared with conventional chemotherapy for either metastatic breast cancer or as adjuvant therapy for poor-prognosis primary breast cancer. Ongoing trials addressing these questions have had slow accrual, which may reflect

Correspondence: IF Tannock
Received 23 December 1994; accepted 4 January 1995

the difficulty of doctors in describing, and patients in accepting, RCTs that compare radically different treatments: ABC *vs* XYZ is much easier. Slevin *et al.* did not inform us of the type of RCT presented in their survey, but one wonders if their patients would have been as accepting of an RCT that compared different modalities of treatment (e.g. surgery *vs* radiation and chemotherapy) or treatments with major differences in possible side-effects.

What can be cone to encourage recruitment into important RCTs? I have the following suggestions.

(1) There should be greater selection of important RCTs and rejection of unimportant ones. Some national or international level of review should determine whether an RCT truly addresses controversy and whether it has a high probability of changing practice. Expert physician surrogates might be used: 'Would you be willing to enter this trial if you had the disease?' Novel end points such as quality of life and cost-effectiveness should also be encouraged.

(2) Trials should be kept as simple as possible and allow some flexibility in evaluating strategies. This will allow the participation of more physicians, thus facilitating accrual and increasing the relevance of trial results to routine oncologic practice (Yusuf *et al.*, 1990). This strategy has been employed in designing the AXIS trial for rectal cancer (Gray *et al.*, 1991), although the investigators may have underestimated the conceptual barrier of using intraportal 5-FU instead of the more familiar intravenous 5-FU as one of the experimental treatments.

(3) Participants in most trials are primarily Caucasian, with higher than average levels of education. Strategies are required to encourage the participation of under-represented ethnic and socioeconomic groups. This will also improve the generalisability of trial results.

(4) A combination of carrot and stick might encourage physicians to approach patients about important RCTs. Participation should be an expected component of medical practice, as is maintaining clinical competence and continuing medical education; it should assume greater emphasis in undergraduate and postgraduate medical education, and in advancement and promotion. Failure to participate should require justification (Segelov *et al.*, 1992).

(5) Those who fund health care, be this private or public, should be educated that good clinical research is cheaper than the uncritical adoption of unproven treatments. There is no better example than the preposterous use of high-dose chemotherapy and autologous stem cell rescue for breast cancer throughout the United States, (Antman *et al.*, 1994) with no evidence of improved survival. Some insurance companies will fund the procedure only as part of an RCT. Although this raises ethical questions, it is evident that resources for new treatments are limited, and it is cost-effective to use these resources for rigorous evaluation of new therapies with potential value, and for the deployment of only these that convey clinical benefit. The medical bywords of the 1990s are 'evidence-based medicine': improvement of therapy *and* containment of cost can find common ground here. Those who fund health care should recognise that evidence depends on research and that better and more cost-effective medicine depends on RCTs that address important questions.

**Acknowledgements**
I thank Drs SA McLachlan and M Stockler for their constructive comments.

**References**

ANTMAN KS, ARMITAGE JO, HOROWITZ MM AND ROWLINGS PA. (1994). Autotransplants for breast cancer in North America. *Proc. Am. Soc. Clin. Oncol.*, **13**, 67.

CHLEBOWSKI RT AND LILLINGTON LM. (1994). A decade of breast cancer clinical investigation: results as reported in the Program Proceedings of the American Society of Clinical Oncology. *J. Clin. Oncol.*, **12**, 1789–1795.

EARLY BREAST CANCER TRIALISTS' COLLABORATIVE GROUP. (1992). Systemic treatment of early breast cancer by hormonal, cytotoxic, or immune therapy. 133 randomized trials involving 31,000 recurrences and 24,000 deaths among 75,000 women. *Lancet*, **339**, 1–15, 71–75.

FREEDMAN B. (1987). Equipoise and the ethics of clinical research. *N. Engl. J. Med.*, **317**, 141–145.

GRAY R, JAMES R, MOSSMAN J AND STENNING S. (1991). AXIS–a suitable case for treatment. *Br. J. Cancer*, **63**, 841–845.

MACKILLOP WJ, PALMER MJ, O'SULLIVAN B, WARD GK, STEELE, R AND DOTSIKAS G. (1989). Clinical trials in cancer: the role of surrogate patients in defining what constitutes an ethically acceptable clinical experiment. *Br. J. Cancer*, **59**, 388–395.

MOORE M, O'SULLIVAN B AND TANNOCK IF. (1990). Are treatment strategies of urologic oncologists influenced by the opinions of their colleagues? *Br. J. Cancer*, **62**, 988–991.

SEGELOV E, TATTERSALL MHN AND COATES AS. (1992). Redressing the balance – the ethics of *not* entering an eligible patient on a randomized clinical trial. *Ann. Oncol.*, **3**, 103–105.

SLEVIN M, MOSSMAN J, BOWLING A, LEONARD R, STEWARD WA, HARDER P, McILLMURRAY M AND THATCHER N. (1995). Volunteers or victims: patients' views of randomised clinical trials. *Br. J. Cancer*, **71**, 1264–1268.

TAYLOR KM, MARGOLESE RG AND SOSKOLNE CL. (1984). Physicians' reasons for not entering eligible patients in a randomized clinical trial of surgery for breast cancer. *N. Engl. J. Med.*, **310**, 1363–1367.

YUSUF S, HELD P, TEO KK AND TORETSKY ER. (1990). Selection of patients for randomized controlled trials: implications of wide or narrow eligibility criteria. *Stat. Med.*, **9**, 73–86.